# Brainstem Raphe Echogenicity and Insomnia in Type 2 Diabetes: An Exploratory Cross-Sectional Study

**DOI:** 10.3390/life16020298

**Published:** 2026-02-09

**Authors:** Maarja Randväli, Kaja Mädamürk, Jekaterina Šteinmiller, Toomas Toomsoo

**Affiliations:** 1 Department of Nursing, Tallinn Health University of Applied Sciences, 13418 Tallinn, Estonia; jekaterina.steinmiller@ttk.ee; 2 School of Natural Sciences and Health, Tallinn University, 10120 Tallinn, Estonia; kaja.madamurk@tlu.ee (K.M.); toomas.toomsoo@tlu.ee (T.T.)

**Keywords:** type 2 diabetes, transcranial sonography, raphe nuclei, depression, insomnia, sleep-related symptoms, brainstem echogenicity, exploratory study

## Abstract

Background: Type 2 diabetes mellitus (T2DM) is associated with increased vulnerability to depression and other affective disturbances, potentially mediated by neurobiological alterations in the serotonergic brainstem raphe nuclei. This study examined whether raphe hypoechogenicity, assessed by transcranial sonography, is associated with depressive, anxiety, and sleep-related symptoms in individuals with T2DM, and whether such alterations are linked to diabetes-related complications. Methods: This cross-sectional study included 230 participants with T2DM and non-diabetic controls. Raphe echogenicity was assessed using transcranial sonography (TCS), and mental health outcomes were measured with the Patient Health Questionnaire 9 (PHQ-9) and the Emotional Well-Being Questionnaire (EWQ). To address demographic imbalance, analyses were repeated in a propensity score–adjusted subsample (n = 89). Results: Raphe hypoechogenicity was associated with higher insomnia scores (EWQ6; β = 0.67, *p* = 0.01); however, this association was attenuated to non-significance after adjustment for sleep medication use and did not survive correction for multiple comparisons, and no associations were observed with PHQ-9 or other EWQ subscales. The participants with hypoechogenic raphe also exhibited a higher prevalence of other diabetes-related complications (32% vs. 7%, *p* = 0.03). Conclusions: In this exploratory cross-sectional sample, reduced raphe echogenicity was not associated with overall depressive or anxiety symptom severity, but was associated with higher self-reported sleep-related symptom burden. However, this association was not robust to adjustment for sleep medication use or to correction for multiple comparisons. These findings are hypothesis-generating and require replication in larger, longitudinal and medication-naive cohorts using standardized sleep instruments.

## 1. Introduction

Type 2 diabetes mellitus (T2DM) is a chronic metabolic disorder that, beyond its well-known physical complications, frequently impairs mental health [1,2]. Several studies have demonstrated that individuals with diabetes exhibit a significantly higher prevalence of depression compared to the general population—estimated to be up to twice as high [1,3]. In addition, individuals with diabetes are at an elevated risk of developing anxiety disorders [4]. Mental health disorders not only complicate self-management and adherence to treatment regimens, but can also exacerbate disease progression [5]. Untreated mental health issues in patients with diabetes can lead to poor treatment compliance, suboptimal glycemic control, increased risk of complications and, ultimately, a diminished quality of life [1,5].

The relationship between depression and T2DM is likely bidirectional: while T2DM increases the risk of developing depressive symptoms, depression itself also heightens the risk of diabetes onset [6,7]. Several biological mechanisms for this relationship have been proposed, including chronic low-grade inflammation, hormonal dysregulation, and impaired insulin signaling in the brain [8]. A recent systematic review demonstrated that biological, psychological, and social risk factors—such as dysglycemia, obesity, comorbidities, and lack of psychosocial support—jointly contribute to depression and cognitive dysfunction in T2DM. These findings emphasize the multidimensional vulnerability of patients with diabetes, providing a rationale for investigating potential neurobiological correlates of these disturbances [9]. Notably, insulin receptors are distributed across multiple brain regions involved in emotion regulation, such as the ventral tegmental area, amygdala, and brainstem raphe nuclei [10].

Given that insulin resistance is a hallmark of T2DM and affects both peripheral and central pathways, alterations in insulin signaling may disrupt serotonergic and stress-related neurocircuits, potentially contributing to depressive symptomatology in diabetic populations [11,12]. Furthermore, diabetes-related complications may also encompass central nervous system (CNS) damage, which has been implicated in the pathophysiology of mood disorders [13]. These findings collectively suggest that T2DM may exert direct neurobiological effects on brain structures responsible for mood regulation, including serotonergic nuclei. Beyond neurobiological alterations, individuals with T2DM also face substantial psychosocial and emotional challenges that complicate adaptation to the disease. Our previous work highlighted the multidimensional nature of coping and psychological adjustment in this population, emphasizing that emotional well-being is a critical yet often underrecognized component of diabetes management [14].

Understanding these psychological, neurobiological, and behavioral interactions is also highly relevant for healthcare practice. Individuals with T2DM experience elevated rates of depression, anxiety, fatigue, and sleep disturbances, all of which impair medication adherence, glucose monitoring, and lifestyle modification [15,16]. Early recognition of emotional distress is therefore essential in routine care as these symptoms meaningfully shape diabetes self-management and the risk of complications. Moreover, advances in neurobiological research—which have demonstrated links between central insulin signaling, serotonergic pathways, and mood regulation—highlight that emotional symptoms in T2DM often have a biological basis rather than being merely psychological reactions [17]. Incorporating such knowledge into clinical practice strengthens person-centered, stigma-reducing approaches and supports timely identification of patients who may benefit from psychosocial assessment or specialized referral.

Diabetes specialist nurses play a central yet underrecognized role in detecting psychosocial distress among individuals with T2DM. In many healthcare systems, these professionals conduct the majority of routine follow-up consultations and are ideally positioned to identify early signs of sleep disturbances, fatigue, and emotional difficulties. However, systematic assessment of sleep quality remains uncommon in diabetes nursing practice, partly due to time constraints and partly due to the assumption that such symptoms fall outside the scope of metabolic care. Establishing the neurobiological basis of sleep disturbances in T2DM may provide a rationale for integrating sleep screening into routine nursing assessments and for developing targeted educational interventions [16].

The raphe nuclei are clusters of neurons located in the brainstem, which serve as the primary source of serotonergic innervation to the forebrain. The dorsal raphe nucleus in particular plays a central role in regulating mood, anxiety, and the sleep–wake cycle [18]. Neuroimaging studies have revealed that individuals with major depressive disorder (MDD) often show structural and functional changes in the raphe region [19,20,21]. For example, transcranial sonography (TCS) has demonstrated that the brainstem raphe nuclei in patients with severe depression appears hypoechogenic—i.e., show reduced ultrasound reflectivity compared to healthy controls. This finding has been interpreted as a potential marker of neurochemical or structural disturbance in the serotonergic system [22]. Similar patterns of reduced raphe echogenicity have also been observed in patients with neurological diseases accompanied by depression, including Parkinson’s disease and migraine [23,24].

Taken together, these data raise the possibility that T2DM-associated alterations in brain insulin signaling and mood-related neural pathways, including the raphe nuclei, may underlie the increased vulnerability to depression observed in diabetic populations. Identifying objective neuroimaging correlates of mental health disturbances in T2DM is critical for improving early detection, tailoring interventions, and understanding the neurobiological underpinnings of diabetes-related affective disorders. Given the known association between raphe hypoechogenicity and depression in other populations, we hypothesized that T2DM patients with hypoechogenic raphe nuclei would exhibit higher scores on measures of depression (PHQ-9), anxiety, and sleep disturbances (EWQ subscales) compared to those with normal raphe echogenicity and non-diabetic controls. For the purpose of statistical inference, insomnia symptoms assessed using the EWQ6 subscale were defined a priori as the primary outcome, whereas depressive and anxiety-related symptoms were analysed as secondary exploratory outcomes. As a secondary aim, we explored whether raphe alterations were associated with diabetes-related complications and metabolic indicators.

## 2. Materials and Methods

### 2.1. Study Design

This study employed a cross-sectional observational design and was conducted between February 2023 and March 2024 at two medical centers in Estonia: Confido Medical Centre and North Estonia Medical Centre. Both sites were selected due to their diverse outpatient populations with T2DM and their experience with transcranial ultrasound and healthcare providers. The study followed the STROBE (Strengthening the Reporting of Observational Studies in Epidemiology) guidelines to ensure rigorous methodological quality [25].

### 2.2. Study Sample

**Figure 1:**
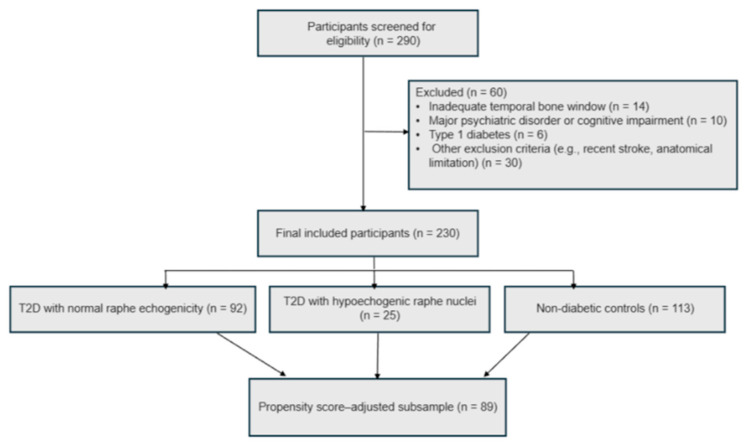
Flowchart of participant selection and inclusion process. Note: The propensity score–balanced subsample (n = 89) includes participants within the region of common support of the estimated propensity score distributions, ensuring comparable distributions of age, sex, and educational level across groups. This approach was used to reduce confounding due to baseline sociodemographic differences rather than to perform one-to-one matching.

Participants were recruited from endocrinology outpatient services. Eligible individuals were adults aged 18 years or older with a confirmed diagnosis of T2DM for at least one year and adequate acoustic bone windows for transcranial ultrasound. Exclusion criteria included type 1 diabetes, major psychiatric or cognitive disorders, recent stroke, and anatomical limitations preventing adequate ultrasound imaging. Of the 290 individuals initially screened for eligibility, 230 met the inclusion criteria and completed all study procedures (80% participation rate). The primary reasons for exclusion were inadequate bone window or neurological and psychiatric comorbidities (Figure 1). The final sample consisted of 230 participants, who were divided into three groups: (1) 92 individuals with T2D and normal raphe echogenicity, (2) 25 individuals with T2D and hypoechogenic raphe nuclei, and (3) 113 non-diabetic controls.

Control group participants were recruited primarily from among the spouses or household partners of diabetic participants (approximately 65%), with the remainder drawn from comparable community settings (35%). This strategy was designed to minimize lifestyle- and environment-related confounding factors but it introduced several important constraints. Spouses often share psychosocial stressors, health behaviors, and daily routines, which may attenuate true differences between the diabetic and non-diabetic groups. They may also share genetic predispositions, dietary patterns, and activity levels that increase their own metabolic risk, potentially reducing the distinctiveness of the control group. In addition, the emotional impact of living with or caring for a partner with a chronic illness may influence mental health outcomes in ways not representative of the general non-diabetic population. Consequently, although commonly used in T2DM research, this recruitment approach may limit the external validity of group comparisons, particularly for psychological and behavioral variables.

All participants were fluent Estonian speakers and provided written informed consent. A priori sample size estimation using GPower 3.1 (f = 0.25, α = 0.05, 1 − β = 0.80) indicated that at least 159 participants were required to detect medium effects in between-group comparisons [26]. The achieved sample (N = 230) thus ensured sufficient statistical power. The overall participation rate was 80%, and no systematic dropout bias was observed.

### 2.3. Data Collection

Each participant completed a structured questionnaire assessing sociodemographic data (age, sex, education, and employment status), diabetes-related clinical information (duration of illness, treatment type, hemoglobin A1c [HbA1c], body mass index [BMI], and complications), and medication use (e.g., antidepressants and sleep medications). Mental health data were collected using the Emotional Well-being Questionnaire (EWQ) [27] and Patient Health Questionnaire (PHQ-9) [28].

Non-diabetic status in control participants was verified through self-report and review of medical history without biochemical screening (e.g., fasting glucose or HbA1c), which introduces a potential risk of misclassification. A proportion of control participants were recruited from spouses or household partners, which may further increase the likelihood of shared, unmeasured metabolic or environmental risk factors.

A blinded neurologist performed transcranial sonography to assess raphe echogenicity. The examiner was blinded to participants’ clinical characteristics and questionnaire data. Clinical assessments and questionnaires were conducted in quiet rooms and took approximately 30 to 45 min per participant. Although participants were recruited at two medical centres, all transcranial sonography acquisitions were centralised and performed at a single site by the same examiner using the same ultrasound device and protocol, ensuring uniform acquisition conditions across participants. All transcranial sonography examinations were performed by a single experienced examiner, as no other certified specialists in this imaging modality were available in Estonia at the time of the study. The use of a single rater precluded formal inter-rater reliability assessment; however, it ensured complete methodological consistency across all participants. All examinations were conducted in the same dedicated examination room using the same ultrasound device equipped with a 2 MHz probe, following standard midbrain axial imaging protocols through the temporal bone window. Device presets (gain, depth, and dynamic range) were kept constant across examinations, and midbrain axial planes were acquired following the same predefined checklist to ensure protocol adherence. This standardized approach minimized potential variability related to equipment, environmental conditions, or operator technique.

Classification followed previously established protocols [20] in which raphe echogenicity is rated on visual inspection of the midbrain at the level of the aqueduct. A rating of ‘normal’ requires a continuous, well-defined echogenic line extending across the midline with a signal intensity comparable to that of surrounding structures, whereas ‘hypoechogenic’ is defined as an interrupted, barely visible, or completely absent midline signal. To minimize subjectivity, the raters were provided with reference images during training sessions.

### 2.4. Measurements

Psychological symptoms were assessed using two self-report instruments. Depressive symptom severity was evaluated with the Patient Health Questionnaire-9 (PHQ-9), a widely used and internationally validated screening tool for depressive symptoms. In addition, participants completed the Emotional Well-Being Questionnaire (EWQ), a nationally validated instrument used in Estonian clinical and research settings to assess the frequency of emotional and psychosocial symptoms over the preceding month.

Mental health was assessed with the PHQ-9, a nine-item self-report tool for screening depressive symptoms based on Diagnostic and Statistical Manual of Mental Disorders, Fourth Edition (DSM-IV) criteria [28]. The total score ranges from 0 to 27; in this study, the sum score was used in the analyses (range: 0–19). The EWQ (Emotional Well-being Questionnaire) developed by the Psychiatry Clinic of the University of Tartu is a self-report symptom screening instrument used in Estonia to assess the frequency of emotional and psychosocial symptoms over the preceding month, including symptoms related to anxiety, depression, fatigue, and sleep. The EWQ is not intended to establish psychiatric diagnoses but to capture symptom burden across multiple affective domains. It consists of six subscales: depression (EWQ1), generalized anxiety (EWQ2), panic disorder (EWQ3), social anxiety (EWQ4), asthenia or mental exhaustion (EWQ5), and insomnia (EWQ6). Each item is rated on a five-point Likert scale (0 = not at all, 4 = constantly), reflecting symptom frequency over the past month [27]. The psychometric properties of the EWQ have recently been evaluated in an Estonian type 2 diabetes cohort and demonstrated acceptable reliability (Cronbach’s α = 0.79) and convergent validity with the PHQ-9 (r = 0.65), supporting its suitability for assessing emotional well-being in this population [29]. However, the EWQ is a regionally validated instrument, and its use limits direct international comparability with studies employing globally established psychiatric or sleep-specific measures. Accordingly, future studies should incorporate internationally established instruments, such as the Pittsburgh Sleep Quality Index or the Insomnia Severity Index, to enhance comparability. The EWQ6 insomnia subscale includes multiple items assessing sleep-related complaints, whereas the PHQ-9 contains a single sleep-related item (item 3). Differences observed between these measures may therefore reflect differences in item coverage and measurement of sensitivity rather than true symptom-specific or biological differences. Accordingly, findings based on EWQ subscales should be interpreted as exploratory and instrument dependent.

Diabetes-related clinical data included disease duration (≤10 years, 11–19 years, and ≥20 years), HbA1c levels (<6.5%, 6.5–7.0%, 7.1–7.5%, and >7.5%), treatment type (oral vs. insulin-based), and presence of complications such as retinopathy, neuropathy, nephropathy, trophic ulcers, foot complications, hypoglycemia, and other diabetes-related conditions. Additional variables included history of stroke, myocardial infarction, arterial hypertension, cardiac arrhythmias, and dyslipidemia. Medication usage data were collected for beta-blockers, antidepressants, and prescribed sleep medications used during the previous month. BMI was measured on site and categorized as underweight (<18.5), normal (18.5–24.9), overweight (25.0–29.9), or obese (≥30.0). For participants aged 65 years or older, BMI was interpreted according to age-appropriate reference values (normal: 24–29 kg/m^2^). Echogenicity of the raphe nuclei was the primary imaging variable and served as the main grouping factor within the T2D sample. This variable was assessed independently of mental health outcomes to minimize observer bias. Age, sex, BMI, diabetes duration, and employment status were included as covariates in all regression analyses as these factors are known to influence both metabolic control and affective symptomatology.

Post hoc power analysis indicated that the propensity-adjusted subsample (n = 89) retained approximately 80% power to detect medium-sized between-group differences in EWQ6 insomnia scores (Cohen’s f = 0.30) at α = 0.05, confirming that the analysis was adequately powered to detect effects of moderate magnitude (calculated using G*Power 3.1).

### 2.5. Demographic and Clinical Background

Regarding demographic data (Table 1), the three groups had similar proportions of persons with secondary and tertiary education. The groups had somewhat different proportions of men and women, but this difference did not reach statistical significance (*p* = 0.07). However, there was slightly more retired persons in Group 2 and fewer in Group 3 [χ^2^(2) = 26.02, *p* < 0.001]. Accordingly, persons in Group 2 were more likely to be 65 years or older [χ^2^(2) = 26.17, *p* < 0.001], and the average age differed significantly between the groups. Group 3 was the youngest (M = 58.58, SD = 9.34), Group 1 was intermediate (M = 63.65, SD = 9.60), and Group 2 was the oldest (M = 70.04, SD = 8.41).

To further address baseline demographic imbalances—particularly in age and employment status—a propensity score adjustment was applied. Propensity scores were computed using multinomial logistic regression with age, sex, and education as predictors of group membership. The participants with overlapping propensity distributions across all three groups were retained, resulting in a matched subsample of 89 individuals (Group 1: n = 29; Group 2: n = 25; Group 3: n = 35) (Table 2). The subsample showed no significant demographic differences across groups (all *p* > 0.25), indicating adequate covariate balance after adjustment. This propensity score-adjusted subsample was used in the subsequent sensitivity analyses to confirm the robustness of the main findings regarding raphe echogenicity and depressive symptom measures.

### 2.6. Statistical Analysis

All analyses were based on complete-case data; missing values (<3% per variable) were inspected for randomness and handled by listwise deletion. Statistical analyses were conducted using IBM SPSS Statistics, version 24, and Mplus, version 8.6 [30]. Descriptive statistics (means, standard deviations, frequencies, and percentages) were calculated to summarize the sociodemographic and clinical variables. Chi-square (χ^2^) tests were used to compare the categorical variables between the three groups [31] (T2D with normal raphe nuclei, T2D with hypoechogenic raphe nuclei, and non-diabetic controls), including sex, age group, BMI categories, medical history (e.g., stroke and arrhythmias), and medication use. For continuous variables, including PHQ-9 scores and EWQ subscale scores, between-group comparisons were performed using one-way analysis of variance (ANOVA). When statistically significant main effects were found, Bonferroni-corrected post hoc tests were used to identify pairwise group differences. Effect sizes were calculated using eta squared (η^2^) [32]. Additional χ^2^ tests were conducted to examine associations between raphe echogenicity and antidepressant or sleep medication use. To determine whether having T2D and hypoechogenic raphe nuclei predicted PHQ-9 and EWQ subscale scores after controlling for potential confounders (age, sex, and BMI), multiple linear regression analyses were performed using maximum likelihood estimation with robust standard errors. Analyses conducted in the full sample (N = 230) were considered primary. Propensity-score–restricted analyses were performed as sensitivity analyses to assess robustness of findings to covariate imbalance. Results from both approaches are presented to allow comparison. As a sensitivity check, all main models were re-estimated within a propensity score–balanced subsample (n = 89). To reduce confounding due to group differences in age, sex, and educational level, this subsample was created by restricting analyses to participants within the region of common support of the estimated propensity score distributions. This approach aimed to retain participants with overlapping sociodemographic characteristics across groups rather than performing one-to-one matching. Comparable results across analyses confirmed the robustness of the findings. Statistical significance was set at *p* < 0.05 (two-tailed).

This study examined seven outcome measures (PHQ-9 and the six EWQ subscales), which raises the possibility of inflated Type I errors. Given the exploratory nature of the work and the limited existing research on raphe echogenicity in type 2 diabetes, no formal multiple-comparison correction was applied in the primary analyses as the aim was to generate hypotheses rather than to provide confirmatory evidence. This approach increases the risk of false-positive findings, so the results should therefore be interpreted with appropriate caution. Notably, if a conservative Bonferroni-adjusted threshold were applied (α = 0.05/7 = 0.007), the observed association with insomnia (*p* = 0.01) would not meet statistical significance. To support transparent interpretation, we report uncorrected *p*-values alongside effect sizes, enabling readers to evaluate both the statistical and practical relevance of the results. All findings should be regarded as preliminary until replicated in independent, prospectively designed studies that pre-specify primary outcomes and apply appropriate corrections for multiplicity.

## 3. Results

**Figure 2:**
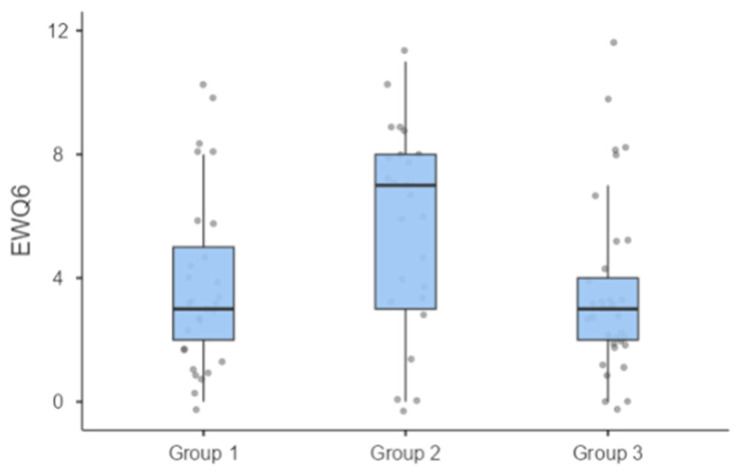
Distribution of insomnia scores (EWQ6) across study groups in the propensity score-matched subsample. Note: Boxes represent interquartile ranges (IQRs), horizontal lines indicate medians, and whiskers denote minimum and maximum values. Individual dots represent participant scores. *p* < 0.01 for Group 2 versus the other groups.. EWQ6 = item assessing insomnia from the Emotional Well-being Questionnaire; analysis based on the propensity score-matched subsample.

Group comparisons within the propensity score-adjusted subsample revealed no significant differences across groups in most EWQ domains or PHQ-9 scores. However, the participants with T2D and hypoechogenic raphe nuclei (Group 2) reported significantly higher insomnia symptoms (EWQ6) compared to both the T2D group with normal raphe nuclei and non-diabetic controls (*p* = 0.01, η^2^ = 0.10) (Table 3). While reduced raphe nucleus echogenicity was associated with higher scores on the EWQ-6 sleep-related subscale, no corresponding association was observed for the PHQ-9 sleep item. The modest correlation between these two indicators suggests that they may capture partially overlapping but non-identical aspects of sleep-related symptom reporting. The participants with raphe hypoechogenicity (Group 2) exhibited significantly higher insomnia scores compared with the other groups (*p* < 0.01) (Figure 2).

### 3.1. Associations with Depressive and Emotional Well-Being Outcomes

Multiple linear regression models were used to examine associations between raphe echogenicity and depressive and emotional well-being outcomes after adjustment for age, sex, BMI, and employment status (Table 4). Analyses conducted in the propensity score-adjusted subsample are presented as sensitivity analyses to assess robustness of the findings. The results showed that hypoechogenic raphe nuclei (Group 2) were associated with higher insomnia symptom scores (EWQ6) (β = 0.67, 95% CI [0.16, 1.18], *p* = 0.01) after adjustment for covariates. BMI was also associated with insomnia symptoms (β = 0.27, 95% CI [0.02, 0.52], *p* = 0.04), suggesting that both raphe echogenicity status and body weight were related to sleep-related complaints. After additional adjustment for prescribed sleep medication use, the association between raphe hypoechogenicity and EWQ6 scores was attenuated and no longer statistically significant (β = 0.47, SE = 0.26, 95% CI [−0.04, 0.98], *p* = 0.07). Using sleep medication was a significant predictor of insomnia symptoms (β = 0.58, SE = 0.25, 95% CI [0.09, 1.07], *p* = 0.02), BMI remained as a significant predictor of insomnia symptoms (β = 0.27, SE = 0.09, 95% CI [0.09, 0.45], *p* = 0.01). Age (β = −0.04, SE = 0.11), being in the control group (β = −0.12, SE = 0.21), gender (β = −0.01, SE = 0.22), and employment status (β = −0.39, SE = 0.29) remained non-significant predictors (*p* > 0.05). Full coefficients and corresponding uncertainty for the fully adjusted model, including sleep medication use alongside all other predictors, are presented in Supplementary Table S1. Adding sleep medication to the model 1 (reported in Table 4) increased the explained variance of insomnia symptoms from r^2^ = 0.17 to 0.23, indicating potential confounding by medication use.

In contrast, depressive symptoms (PHQ-9) were not significantly associated with raphe echogenicity when the covariates were controlled for; however, age (β = −0.30, *p* = 0.04) and female gender (β = 0.50, *p* = 0.02) were independent predictors of higher PHQ-9 scores. No other EWQ subscales were related to raphe echogenicity after adjustment for covariates. Simple bivariate correlations (Table 5) indicated modest associations between hypoechogenic raphe nuclei and higher PHQ-9 and EWQ6 scores (r = 0.19 and r = 0.18, respectively; both *p* < 0.05), which were directionally consistent with the adjusted analyses but should be interpreted cautiously given the exploratory design.

### 3.2. Cardiometabolic Comorbidities and Medication Use

In the propensity score-adjusted subsample, no significant group differences were observed in the prevalence of stroke, myocardial infarction, hypertension, cardiac arrhythmias, dyslipidemia, or beta-blocker use (all *p* > 0.25) (Table 6). However, a clear trend was noted for antidepressant use, which was more frequent among the participants with T2D and hypoechogenic raphe nuclei (16%) compared to the two other groups (10% and 0%), though this difference did not reach statistical significance (*p* = 0.06). A statistically significant group difference emerged for use of prescribed sleep medication, which was reported by 40% of the participants with hypoechogenic raphe nuclei compared with only 7% in both the T2D group with normal raphe nuclei and the control group [χ^2^(2) = 8.83, *p* = 0.01]. Mean BMI did not differ significantly between groups (*p* = 0.21).

### 3.3. Diabetes-Related Treatment and Complications

Table 7 presents the distribution of diabetes-related treatment characteristics and complications within the propensity score-adjusted subsample. Chi-square tests were used to determine the difference between expected and observed counts; due to the small sample size, Fisher’s Exact Test *p*-value was used to estimate statistical significance. No significant group differences were observed in diabetes treatment type, use of oral versus insulin therapy, or prevalence of hypertension, retinopathy, nephropathy, neuropathy, or hypoglycemia (all *p* > 0.10). A non-significant trend was found for trophic ulcers, which were only reported among the participants with hypoechogenic raphe nuclei (12%) but not in the group with normal raphe nuclei (*p* = 0.09).

The category “other diabetes-related complications” comprised various less-common comorbidities recorded in medical charts, including cardiovascular, endocrine–metabolic, neurological, and respiratory conditions (e.g., heart failure, thyroid dysfunction, epilepsy, and sleep apnea). These were significantly more frequent among participants with hypoechogenic raphe nuclei (32%) than those with normal raphe nuclei (7%) [χ^2^(1) = 5.33, *p* = 0.03]. Within this category, sleep apnea was reported in 4 participants (14%) in Group 1 (T2D with normal raphe nuclei) and 8 participants (32%) in Group 2 (T2D with hypoechogenic raphe nuclei) (Table 7). Obstructive sleep apnea status was based on medical history documentation and was not assessed using a standardized sleep instrument; therefore, results are reported descriptively. These findings suggest that the subgroup of patients with hypoechogenic raphe nuclei may have a slightly greater overall burden of diabetes-related complications, although most specific complications did not differ significantly between groups.

## 4. Discussion

This study explored the relationship between midbrain raphe echogenicity and affective, emotional, and sleep-related symptoms in individuals with type 2 diabetes. While hypoechogenicity of the raphe nuclei was not associated with overall depressive or anxiety symptom severity, a distinct association emerged with sleep-related symptoms, particularly insomnia. In addition, raphe hypoechogenicity was more frequently observed among participants with a higher burden of diabetes-related complications. Together, these findings suggest a possible and potentially non-linear pattern involving raphe echogenicity, symptom expression, and disease burden, which requires cautious interpretation and further investigation.

### 4.1. Interpretation of Findings

Although reduced raphe nucleus echogenicity has been reported in depressive and neurological disorders, its biological basis remains incompletely understood. While serotonergic involvement represents one plausible hypothesis, alternative mechanisms—including microvascular changes related to diabetes, neuroinflammatory processes, age-related structural alterations, and impaired glymphatic clearance—should also be considered. As transcranial sonography reflects tissue echogenicity rather than specific neurochemical processes, mechanistic interpretations should remain cautious and hypothesis-generating.

The observed association between raphe hypoechogenicity and insomnia may reflect, among several possible mechanisms, serotonergic involvement, given that the midbrain raphe nuclei constitute a major source of projections to sleep-regulatory regions [33,34]. However, this interpretation remains provisional. Transcranial sonography assesses tissue echogenicity rather than neurotransmitter activity, and reduced echogenicity is more likely to index microstructural tissue characteristics—such as gliosis or altered tissue composition—whose biological substrates remain incompletely understood. While raphe hypoechogenicity has been reported in psychiatric and neurodegenerative contexts, the present findings do not indicate symptom-specific neurobiological selectivity. In metabolic disorders, sleep disturbances are plausibly shaped by multiple, interacting influences, including vascular, inflammatory, and medication-related factors, and the cross-sectional design precludes inferences about temporal ordering or causality [35]. Importantly, the association with EWQ-6 should be interpreted cautiously. Differences between EWQ-6 and the PHQ-9 sleep item are more likely to reflect variation in item scope and measurement sensitivity than any biological specificity of raphe nucleus echogenicity for insomnia. In the absence of internationally standardized insomnia-specific instruments, these results should be considered exploratory.

The absence of a significant association with overall depressive severity likely reflects both pharmacological and measurement factors. Antidepressant therapy can alleviate core affective symptoms captured by the PHQ-9 while simultaneously altering sleep architecture, thereby modulating insomnia independently of mood [35]. Furthermore, the PHQ-9 contains only single items for sleep and fatigue, potentially underrepresenting these domains, which are strongly influenced by metabolic factors in T2D. The EWQ subscales may demonstrate higher sensitivity to sleep disturbances in this sample, suggesting that multidimensional instruments may better capture affective symptomatology in metabolic populations. Accordingly, differences between EWQ and PHQ-9 findings should be interpreted as instrument-dependent rather than as evidence of differential neurobiological mechanisms.

Emerging theoretical frameworks have highlighted glymphatic dysfunction as a potential link between sleep disturbance and psychiatric symptoms, particularly in systemic and metabolic conditions [36].

Given the exploratory nature of this study, formal correction was not applied to avoid excessive type II error. Nevertheless, this limitation substantially weakens inferential robustness, and the findings should be interpreted as preliminary signals requiring confirmation in adequately powered confirmatory studies. Notably, adjustment for prescribed sleep medication attenuated the association between raphe hypoechogenicity and insomnia symptoms to non-significance, indicating that the observed relationship was not independent of treatment-related factors and may reflect a more clinically complex sleep disturbance profile rather than insomnia severity per se. The higher prevalence of sleep medication use among participants with raphe nucleus hypoechogenicity represents an important source of confounding. Although statistical adjustment attenuated the observed associations, it cannot fully resolve uncertainty regarding causal directionality. Sleep medication use may reflect greater baseline insomnia severity and could lie on the causal pathway, resulting in residual confounding or overadjustment. Given the cross-sectional design, causal inferences cannot be drawn.

### 4.2. Clinical Implications for Healthcare Practice

The observed link between raphe alterations and insomnia—interpreted as exploratory and instrument-dependent—carries direct relevance for nursing and primary care practice. Diabetes nurses and primary care providers are often the first and most frequent point of contact for individuals with T2D, placing them in a unique position to detect sleep disturbances early. Yet sleep quality is rarely assessed systematically in routine diabetes consultations, where the focus typically remains on glycemic control, medication adjustment, and complication screening.

Sleep disturbances in T2D are not merely quality-of-life concerns; they are frequently associated with impaired self-management behaviors. Poor sleep impairs executive function and motivation, reducing the accuracy of insulin dosing, the consistency of blood glucose monitoring, and adherence to dietary and exercise recommendations. Fatigue and daytime sleepiness may be misattributed to “lifestyle choices” or perceived as inevitable consequences of aging, leading both patients and clinicians to overlook potentially modifiable contributors. Importantly, given the cross-sectional and exploratory nature of the present findings, sleep disturbances should be viewed as clinically relevant signals warranting further assessment rather than as direct consequences of specific neurobiological alterations.

From a practical standpoint, brief validated instruments appropriate to the clinical context—such as the EWQ insomnia subscale in regional settings or internationally established tools like the Insomnia Severity Index—can be integrated into routine diabetes visits with minimal time burden. When significant sleep disturbances are identified, structured referral pathways—to sleep medicine specialists, psychologists, or psychiatrists—may be considered within multidisciplinary diabetes care teams. Additionally, patient education materials could be developed to explain the potential biological connections between diabetes, brain health, and sleep, empowering individuals to report symptoms without shame.

Future clinical guidelines for diabetes care may benefit from explicitly incorporating sleep assessment as a standard component of annual reviews, alongside retinopathy screening, foot examination, and cardiovascular risk evaluation. Such integration would align with the growing recognition that diabetes is a systemic condition affecting not only peripheral organs but also central nervous system function and emotional well-being, while acknowledging that the neurobiological mechanisms linking these domains remain incompletely understood.

### 4.3. Limitations and Future Directions

The category of “other diabetes-related complications” included a small number of participants with documented obstructive sleep apnea. Sleep apnea may independently influence sleep symptom reporting and the likelihood of sleep medication use and could therefore contribute to residual confounding of the observed associations with sleep-related outcomes. Sensitivity analyses excluding these individuals were not feasible due to insufficient numbers, which would result in unstable estimates. This represents a limitation of the present study. Future research should systematically assess sleep apnea using validated screening instruments or polysomnography and account for its potential confounding effects.

A further limitation relates to the recruitment of a proportion of control participants from spouses or household partners. While this approach may reduce socioeconomic heterogeneity between groups, it may also introduce shared environmental or lifestyle factors that could attenuate or obscure group differences. This should be considered when interpreting comparisons between individuals with type 2 diabetes and controls. A further limitation is that non-diabetic status in control participants was based on self-report and review of medical history rather than biochemical testing. Undiagnosed dysglycemia in controls cannot be excluded and may have attenuated between group differences. The use of propensity-score restriction substantially reduced the analytic sample size (from N = 230 to N = 89), limiting statistical power and increasing susceptibility to unstable effect estimates. Findings derived from restricted samples should therefore be interpreted as supportive sensitivity analyses rather than as primary evidence.

Future work should prioritize rigorous replication in multiple international cohorts using pre-registered protocols, corrected significance thresholds, and standardized TCS procedures to establish reproducibility. Studying medication-naïve patients at diabetes diagnosis and following them after treatment initiation to determine whether pharmacological agents modify raphe echogenicity or sleep trajectories is equally important. Longitudinal designs with repeated TCS assessments over several years are needed to clarify the temporal precedence and test whether baseline hypoechogenicity predicts incidental insomnia.

Biological validity should be strengthened through multimodal approaches integrating TCS with structural and functional neuroimaging and relevant biomarkers of serotonergic, inflammatory, and vascular function. Mechanistic studies—including postmortem validation, diabetic animal models, and targeted pharmacological trials—will help to determine the underlying substrates of echogenicity changes. Only after these foundations are established should clinical utility trials be performed to evaluate whether TCS-guided assessment improves risk stratification or care pathways in T2D, and whether it offers added value beyond existing screening tools.

## 5. Conclusions

In this exploratory study, reduced mesencephalic raphe nucleus echogenicity was associated with greater self-reported sleep-related symptom burden in individuals with type 2 diabetes. However, this association was strongly influenced by sleep medication use, attenuated after statistical adjustment, not robust to correction for multiple comparisons, and observed within a small subgroup. These findings should therefore be regarded as hypothesis-generating. Prospective studies using larger samples, internationally standardized sleep assessments, longitudinal designs, and integration of complementary biomarkers are required to clarify the clinical and biological significance of raphe nucleus echogenicity in metabolic populations.

## Figures and Tables

**Table 1:** Demographic characteristics of the study groups.

Variable	Group 1	Group 2	Group 3	df	χ^2^/F	*p*
Gender (M/F)	41 (45%)/51 (55%)	6 (24%)/19 (76%)	36 (32%)/77 (68%)	2	5.33	0.07
Education (secondary/tertiary)	52 (57%)/39 (43%)	13 (52%)/12 (48%)	66 (58%)/47 (42%)	2	0.34	0.84
Employment (employed/retired)	51 (58%)/37 (42%)	5 (21%)/19 (79%)	84 (75%)/28 (25%)	2	26.02	<0.001
Age ≥ 65 years	34 (44%)	19 (76%)	42 (24%)	2	26.17	<0.001
Average age, M (SD)	63.65 (9.60)	70.04 (8.41)	58.58 (9.34)	—	F = 18.22	η^2^ = 0.14

Note. Percentages within each group are presented. Average age is presented as M (SD). Group 1 = Type 2 diabetes with normal raphe echogenicity; Group 2 = Type 2 diabetes with hypoechogenic raphe nuclei; Group 3 = non-diabetic controls.

**Table 2:** Propensity score-adjusted subsample.

Variable	Group 1 (T2D, Normal), n = 29	Group 2 (T2D, Hypoechogenic), n = 25	Group 3 (Controls), n = 35	df	χ^2^/F	*p*
Gender (M/F)	7 (24%)/22 (76%)	6 (24%)/19 (76%)	11 (31%)/24 (69%)	2	0.58	0.75
Education (secondary/tertiary)	17 (59%)/12 (41%)	13 (52%)/12 (48%)	24 (69%)/11 (31%)	2	1.75	0.42
Employment (employed/retired)	5 (17%)/24 (83%)	5 (21%)/19 (79%)	12 (34%)/23 (66%)	2	2.76	0.25
Age ≥ 65 years	25 (86%)	19 (76%)	25 (71%)	2	2.04	0.36
Average age, M (SD)	71.00 (5.98)	70.04 (8.41)	68.14 (5.99)	—	F = 1.49	η^2^ = 0.03

Note. This subsample represents the participants retained after propensity score adjustment for age, sex, and education. Percentages within each group are presented.

**Table 3:** Comparison of depressive and emotional well-being scores across groups in the propensity score-adjusted subsample.

Variable	Group 1	Group 2	Group 3	F	*p*	η^2^	η^2^ 95% CI
EWQ1—Depression	5.76 (5.59)	7.40 (7.33)	6.17 (4.93)	0.56	0.58	0.01	0.00–0.08
EWQ2—Generalized anxiety	5.83 (4.49)	6.48 (5.04)	6.17 (4.29)	0.14	0.87	0.00	0.00–0.04
EWQ3—Panic disorder	1.14 (2.18)	1.72 (2.17)	0.89 (1.95)	1.18	0.31	0.03	0.00–0.11
EWQ4—Social anxiety	0.59 (0.87)	0.48 (1.30)	0.51 (0.85)	0.08	0.92	0.00	0.00–0.03
EWQ5—Asthenia	5.21 (3.16)	6.52 (3.63)	5.86 (3.47)	0.99	0.38	0.02	0.00–0.10
EWQ6—Insomnia	3.72 (2.83)	5.72 (3.26)	3.49 (2.82)	4.71	**0.01**	**0.10**	**0.00–0.22**
PHQ-9—Depressive symptoms	5.38 (3.48)	7.40 (5.21)	5.31 (3.07)	2.50	0.09	0.06	0.00–0.17

Note. Values represent the mean (M) and standard deviation (SD). Analyses conducted within the propensity score-adjusted subsample (n = 89). One-way ANOVA was used for between-group comparisons. Bold values indicate statistical significance at *p* < 0.05. Group 1 = Type 2 diabetes with normal raphe echogenicity; Group 2 = Type 2 diabetes with hypoechogenic raphe nuclei; Group 3 = non-diabetic controls.

**Table 4:** Multiple regression analyses examining associations with depressive and emotional well-being outcomes within the propensity score-adjusted subsample.

Dependent Variable	Group 2 β (SE)	Group 3 β (SE)	Age β (SE)	BMI β (SE)	Gender β (SE)	Employment β (SE)	r^2^
EWQ1—Depression	0.18 (0.28)	0.04 (0.25)	−0.20 (0.18)	0.15 (0.29)	0.29 (0.26)	0.26 (0.45)	0.04
EWQ2—Generalized anxiety	0.07 (0.27)	0.07 (0.25)	**−0.17 (0.16)**	0.16 (0.14)	0.37 (0.25)	0.36 (0.35)	0.08
EWQ3—Panic disorder	0.17 (0.29)	−0.12 (0.24)	0.07 (0.11)	−0.12 (0.01)	−0.01 (0.24)	0.06 (0.24)	0.02
EWQ4—Social anxiety	−0.11 (0.31)	−0.11 (0.23)	−0.21 (0.16)	0.12 (0.12)	−0.33 (0.26)	0.13 (0.28)	0.06
EWQ5—Asthenia	0.39 (0.27)	0.22 (0.24)	0.04 (0.15)	0.10 (0.11)	0.36 (0.22)	−0.41 (0.38)	0.17
EWQ6—Insomnia	**0.67 (0.26)**	−0.04 (0.22)	−0.03 (0.12)	**0.27 (0.13)**	0.11 (0.23)	0.19 (0.32)	0.17
PHQ-9—Depressive symptoms	0.42 (0.29)	−0.06 (0.23)	**−0.30 (0.17)**	0.01 (0.13)	**0.50 (0.22)**	0.24 (0.44)	0.15

Note. Separate linear regression models were estimated for each dependent variable. Group 1 (T2D with normal raphe nuclei) served as the reference category. Models were adjusted for age, sex, BMI, and employment status and do not include sleep medication use. β coefficients are standardized estimates with standard errors (SE). r^2^ indicates the proportion of variance explained by each model. Analyses were conducted in the propensity score-adjusted subsample (n = 89). Bold coefficients indicate *p* < 0.05. Group 2 = T2D with hypoechogenic raphe nuclei; Group 3 = non-diabetic controls.

**Table 5:** Bivariate correlations between explanatory variables and outcome measures used in regression analyses.

Dependent Variable	Group 2	Group 3	Age	BMI	Gender
EWQ1—Depression	0.14 *	0.04	0.01	0.19 *	0.23 *
EWQ2—Generalized anxiety	0.02	0.13 *	−0.11	0.10	0.28 *
EWQ3—Panic disorder	0.09	0.03	0.01	−0.04	0.07
EWQ4—Social anxiety	−0.03	0.07	−0.14	0.05	0.03
EWQ5—Asthenia	0.11 *	−0.08	0.01	0.24 *	0.08
EWQ6—Insomnia	0.18 *	−0.04	−0.01	0.16 *	0.04
PHQ-9—Depressive symptoms	0.19 *	−0.11	−0.05	0.21 *	0.18 *

Note. Bivariate Pearson correlations were calculated for the variables included in the regression models (Table 3). * *p* < 0.05.

**Table 6:** Cardiometabolic comorbidities and medication use across groups in the propensity score-adjusted subsample.

Variable	Group 1 (T2D, Normal Raphe Nuclei), n (%)	Group 2 (T2D, Hypoechogenic Raphe Nuclei), n (%)	Group 3 (Non-Diabetic Controls), n (%)	df	χ^2^/F	*p*
History of stroke	1 (3%)	2 (8%)	1 (3%)	2	0.97	0.62
History of myocardial infarction	4 (14%)	3 (12%)	3 (9%)	2	0.45	0.80
Arterial hypertension	15 (52%)	11 (44%)	18 (51%)	2	0.14	0.93
Cardiac arrhythmias	12 (41%)	9 (36%)	8 (23%)	2	2.66	0.26
Dyslipidemia	11 (38%)	13 (52%)	14 (40%)	2	1.26	0.53
Use of beta-blockers	11 (38%)	10 (40%)	9 (26%)	2	1.68	0.43
Use of antidepressants in the past 6 months	3 (10%)	4 (16%)	0 (0%)	2	5.52	0.06
Use of prescribed sleep medication in the past month	2 (7%)	**10 (40%)**	2 (7%)	2	**8.83**	**0.01**
BMI, M (SD)	31.11 (3.95)	30.19 (5.12)	29.25 (3.54)	2, 86	F = 1.59	0.21

Note. Percentages are presented within each group. Group 1 = Type 2 diabetes with normal raphe echogenicity; Group 2 = Type 2 diabetes with hypoechogenic raphe nuclei; Group 3 = non-diabetic controls. Bold values indicate statistically significant differences (*p* < 0.05). Analyses were performed using χ^2^ tests for categorical variables and one-way ANOVA for BMI.

**Table 7:** Diabetes-related treatment characteristics and complications in the propensity score-adjusted subsample.

Variable	Group 1 (T2D, Normal Raphe Nuclei), n (%)	Group 2 (T2D, Hypoechogenic Raphe Nuclei), n (%)	df	χ^2^	Fisher’s Exact Test *p*
Type of diabetes treatment (oral medications)	22 (76%)	18 (72%)	1	0.10	0.76
Diabetes treatment type (1 = tablets, 2 = insulin)	15 (88%)	15 (79%)	1	0.56	0.66
Hypertension	23 (79%)	18 (72%)	1	0.39	0.75
Diabetic retinopathy	3 (10%)	6 (24%)	1	1.80	0.28
Diabetic foot	4 (14%)	5 (20%)	1	0.37	0.72
Trophic ulcers	0 (0%)	3 (12%)	1	3.69	0.09
Diabetic nephropathy	4 (14%)	3 (12%)	1	0.04	1.00
Diabetic neuropathy	9 (31%)	9 (36%)	1	0.15	0.78
Other diabetes-related complications	**2 (7%)**	**8 (32%)**	1	**5.33**	**0.03**
Obstructive sleep apnea	4 (14%)	8 (32%)	-	-	-
Hypoglycemia episodes	6 (21%)	7 (28%)	1	0.39	0.75

Note. Percentages are presented within each group. Group 1 = Type 2 diabetes with normal raphe echogenicity; Group 2 = Type 2 diabetes with hypoechogenic raphe nuclei. Bold values indicate statistically significant differences (*p* < 0.05). Other diabetes-related complications included less frequent comorbidities and systemic conditions documented in clinical records such as cardiovascular (heart failure and ischemic disease), endocrine–metabolic (thyroid dysfunction and anemia), neurological (epilepsy, multiple sclerosis, and fibromyalgia), respiratory (sleep apnea and asthma), musculoskeletal (rheumatologic disorders and osteoporosis), chronic inflammatory, and infectious diseases. Sleep apnea status was based on medical history documentation and was not assessed using a standardized sleep instrument; residual confounding by sleep-disordered breathing cannot be excluded. For sleep apnea, no formal between-group statistical testing was performed due to non-standardized ascertainment and small cell counts; values are reported descriptively only.

## Data Availability

The data supporting the findings of this study are available from the corresponding author upon reasonable request, subject to ethical approval and data protection regulations.

## Supplementary Materials

The following supporting information can be downloaded at: https://www.mdpi.com/article/10.3390/life16020298/s1, Table S1: Predictors of insomnia symptoms (EWQ-6) in a propensity score–restricted sample (n = 89), with and without adjustment for prescribed sleep medication use.

## Abbreviations

The following abbreviations are used in this manuscript:

BMI	Body mass index
CNS	Central nervous system
DSM-IV	Diagnostic and Statistical Manual of Mental Disorders, Fourth Edition
EWQ	Emotional Well-Being Questionnaire
HbA1c	Hemoglobin A1c
MDD	Major depressive disorder
PHQ-9	Patient Health Questionnaire-9
T2DM	Type 2 diabetes mellitus
TCS	Transcranial sonography
