# Supplementary material for: Brainstem Raphe Echogenicity and Insomnia in Type 2 Diabetes: An Exploratory Cross-Sectional Study
Source: Life (Basel). 2026 Feb 9;16(2):298. doi: 10.3390/life16020298 (PMC12942166; doi:10.3390/life16020298)
Supplement: Supplementary file 1 [file life-16-00298-s001.zip › life-4106973-supplementary.pdf]

Supplementary Materials

Table S1. Predictors of insomnia symptoms (EWQ-6) in a propensity score–restricted sample (n = 89), with and without adjustment for prescribed sleep medication use.

Predictor	Model 1 β (SE)	95% CI	Model 2 β (SE)	95% CI
Group 2 (T2D, hypoechogenic raphe)	0.67 (0.26)*	[0.16, 1.18]	0.47 (0.26)	[-0.04, 0.98]
Group 3 (non-diabetic controls)	-0.04 (0.22)	[-0.47, 0.39]	-0.12 (0.21)	[-0.53, 0.29]
Age	-0.03 (0.12)	[-0.27, 0.21]	-0.04 (0.11)	[-0.26, 0.18]
BMI	0.27 (0.13)*	[0.02, 0.52]	0.27 (0.09)*	[0.09, 0.45]
Gender	0.11 (0.23)	[-0.34, 0.56]	-0.01 (0.22)	[-0.44, 0.42]
Employment status	0.19 (0.32)	[-0.44, 0.82]	-0.39 (0.29)	[-0.96, 0.18]
Prescribed sleep medication use	-	-	0.58 (0.25)*	[0.09, 1.07]
**Total explained variance of EWQ-6 (r^2)**	0.17		0.23	

*Note: Group 1 (T2D with normal raphe echogenicity) served as the reference category. β coefficients are standardized estimates with standard errors (SE). *p < 0.05.*
